# Poly[aqua­[μ-4-(4-chloro­phen­yl)-2-thioxo-2,3-dihydro­thia­zol-3-olato]­sodium(I)]

**DOI:** 10.1107/S1600536807067736

**Published:** 2008-01-04

**Authors:** Jens Hartung, Janina Bachmann, Ingrid Svoboda, Hartmut Fuess

**Affiliations:** a Fachbereich Chemie, Organische Chemie, Technische Universität Kaiserslautern, Erwin-Schrödinger-Strasse, D-67663 Kaiserslautern, Germany; b Strukturforschung, FB 11, Material- und Geowissenschaften, Technische Universität Darmstadt, Petersenstrasse 23, D-64287 Darmstadt, Germany

## Abstract

The packing of the title compound, [Na(C_9_H_5_ClNOS_2_)(H_2_O)]_*n*_, in the crystal structure occurs by pairwise attachment of +*sc-* and −*sc*-arranged 4-(4-chloro­phen­yl)-2-thioxo-2,3-dihydro­thia­zol-3-olate subunits *via* S to sodium. Water mol­ecules that are bound in the axial position of the distorted octa­hedral coordination octahedron give rise to a stereogenic center at sodium.

## Related literature

For related literature, see: Allen *et al.* (1987 ▶); Hartung *et al.* (1996 ▶, 1999 ▶, 2007 ▶); Nardelli (1999 ▶).
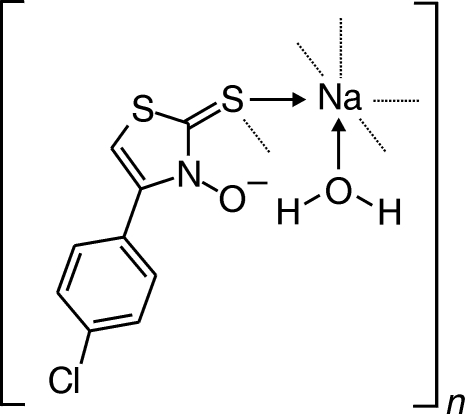


## Experimental

### 

#### Crystal data

[Na(C_9_H_5_ClNOS_2_)(H_2_O)]
*M* *_r_* = 283.72
Orthorhombic, 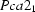

*a* = 39.264 (5) Å
*b* = 4.168 (1) Å
*c* = 7.097 (1) Å
*V* = 1161.4 (4) Å^3^
*Z* = 4
Mo *K*α radiation
μ = 0.71 mm^−1^
*T* = 300 (2) K
0.60 × 0.28 × 0.02 mm

#### Data collection

Oxford Diffraction Xcalibur diffractometer with Sapphire CCD detector
Absorption correction: multi-scan (*CrysAlis RED*; Oxford Diffraction, 2007 ▶) *T*_min_ = 0.677, *T*_max_ = 0.986
5052 measured reflections
2288 independent reflections
1860 reflections with *I* > 2σ(*I*)
*R*_int_ = 0.036

#### Refinement

*R*[*F* ^2^ > 2σ(*F* ^2^)] = 0.081
*wR*(*F* ^2^) = 0.173
*S* = 1.20
2288 reflections
151 parameters
10 restraints
H atoms treated by a mixture of independent and constrained refinement
Δρ_max_ = 0.60 e Å^−3^
Δρ_min_ = −0.83 e Å^−3^
Absolute structure: Flack (1983 ▶), 1009 Friedel pairs
Flack parameter: 0.1 (2)

### 

Data collection: *CrysAlis CCD* (Oxford Diffraction, 2007 ▶); cell refinement: *CrysAlis RED* (Oxford Diffraction, 2007 ▶); data reduction: *CrysAlis RED*; program(s) used to solve structure: *SHELXS97* (Sheldrick, 1997 ▶; program(s) used to refine structure: *SHELXL97* (Sheldrick, 1997 ▶); molecular graphics: *PLATON* (Spek, 2003 ▶) and *ORTEP-3* (Farrugia, 1997 ▶); software used to prepare material for publication: *SHELXL97*.

## Supplementary Material

Crystal structure: contains datablocks I, global. DOI: 10.1107/S1600536807067736/sj2452sup1.cif

Structure factors: contains datablocks I. DOI: 10.1107/S1600536807067736/sj2452Isup2.hkl

Additional supplementary materials:  crystallographic information; 3D view; checkCIF report

## Figures and Tables

**Table 1:** Hydrogen-bond geometry (Å, °)

*D*—H⋯*A*	*D*—H	H⋯*A*	*D*⋯*A*	*D*—H⋯*A*
O2—H2*A*⋯O1^i^	0.88 (5)	1.80 (5)	2.675 (8)	175 (9)
O2—H2*A*⋯N3^i^	0.88 (5)	2.61 (6)	3.434 (9)	156 (8)
O2—H2*B*⋯O1^ii^	0.89 (5)	1.91 (6)	2.770 (8)	164 (9)

Symmetry codes: (i) 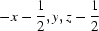; (ii) 
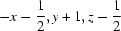
.

**Fig. 1.**
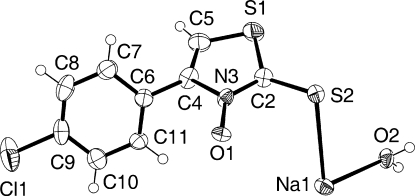
Molecular structure of (I). Displacement ellipsoids are plotted at the 50% probability level.

**Fig. 2.**
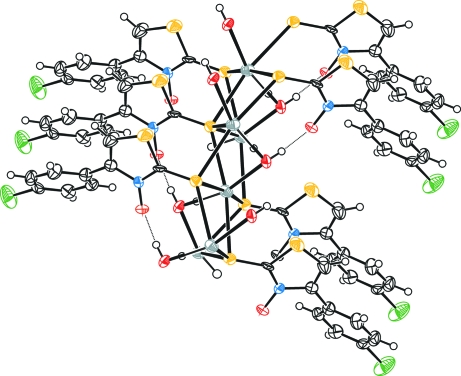
Packing and hydrogen bonding (dashed lines) of (I) in the solid state. S atoms are depicted in yellow, Cl in green, O in red, N in blue, and Na in gray).

## supplementary crystallographic information

### Comment

Neutralization of 4(4-chlorophenyl)-3-hydroxythiazole-2(3*H*)-thione (Hartung *et al.*, 1999) with sodium hydroxide in CH_3_OH furnishes sodium 4-(4-chlorophenyl)-2-thiooxo-2,3-dihydrothiazole-3-olate. The compound crystallizes as monohydrate, (I), from a saturated solution of CH_3_CN/hexane as yellowish prisms. The compound was investigated by X-ray diffraction in order to explore the structural chemistry of an alkali metal thiohydroxamate. The results of the study are summarized in the following section.

Fundamental differences between heterocyclic subunits of sodium 4-(4-chlorophenyl)-2-thiooxo-2,3-dihydrothiazole-3-olate monohydrate, (I) (Fig. 1), and the parent acid, *i.e.*, 4(4-chlorophenyl)-3-hydroxythiazole-2(3*H*)-thione (Hartung *et al.*, 1999) originate from a shortening of the N3—O3 distance from 1.379 (2) Å to 1.329 (8) Å and a lengthening of C2—S2 from 1.684 (2) Å to 1.699 (8) Å. The N3—O1 distance in (I) is closer to values reported for heterocyclic N– oxides than for thiohydroxamic acids (Hartung *et al.*, 1996, 1999, 2007). The C2—S2 bond length lies in between typical values of C—S single and double bonds (Allen *et al.*, 1987, Hartung *et al.*, 1999). The distance C2—N3 [1.33 (1) Å] in sodium salt (I) agrees with the corresponding bond length of 4(4-chlorophenyl)-3-hydroxythiazole-2(3*H*)-thione (Hartung *et al.*, 1999).

The parameters of the thiohydroxamate functional group in (I) are distinctively different from distances reported for 4(4-chlorophenyl)-3-isopropoxy-thiazole-2(3*H*)-thione [N3—O1 = 1.369 (3) Å, C2—S2 = 1.658 (3) Å, C2—N3 = 1.353 (3) Å] and further *N*-alkoxy derivatives thereof (Hartung *et al.*, 1999). One possible explanation for this finding is associated with a significant contribution of the *N*-oxidothiolato formulae for the description of ground state properties of (I) apart from the well established thione resonance formulae. Support for this argumentation comes from sodium atom positioning in the unit cell of (I). The proximity to the metal is in line with 4-(4-chlorophenyl)-2-thiooxo-2,3-dihydrothiazole-3-olate binding as monodentate S-donor ligand to sodium [Na1—S1 = 3.001 (4) Å, S2—Na1A= 2.998 (4) Å, S2—Na1B = 2.958 (4) Å, S2—Na1A = 2.961 (4) Å]. The N-oxide oxygen atom O1 forms hydrogen bonds toward the hydrate water that is attached at either side of the apex of a octahedrally distorted coordination polyhedron at sodium [N3—O1···H2 = 162.2 (3) °, O1···O2A = 2.770 (4) Å] (Figure 2). O1 therefore does not participate in a chelate type of interaction with the metal atom [Na1···O1 = 3.855 (5) A].

The *p*-chlorophenyl substituent is characterized by two different arrangements with respect to the heterocyclic core, *i.e.* positive (+) and negative (-) synclinal [N3—C4—C6—C7 = ± 42 (1) °]. The sodium atom is offset from the heterocyclic plane by Na1—S2—C2—N3 = 53.9 (8) °. A pairwise +*sc* and –sc arrangement of 4-(4-chlorophenyl)-2-thiooxo-2,3-dihydrothiazole-3-olate entities in the equatorial plane in association with a non linearity of the O2—Na1—O2A axis [162.1 (3) °] gives rise to a stereogenic center at sodium (Fig. 2). Chirality thus originates from the packing of individual components of (I) in the solid state.

### Experimental

Sodium hydroxide (40.0 mg, 1.00 mmol, 1 equiv) was added to a solution of 4-(4-chlorphenyl)-3-hydroxythiazol-2(3*H*)-thione (244 mg, 1.00 mmol) in CH_3_OH (5 ml) at 294 K. The reaction mixture was stirred at this temperature for 1.5 h. The volatiles were subsequently removed to afford a yellowish powder. The material was freeze-dried and subsequently dissolved in CH_3_CN/hexane [1/1 (*v*/*v*)]. Yellowish prisms suitable for X-ray diffraction were grown by slowly allowing the solvent to evaporate at 293 K. Analysis calculated for C_9_H_7_ClNNaO_2_S_2_ (283.7 g/mol): C 38.10, H 2.49, N 4.94%; found C 38.15, H 2.39, N 4.92%; ^1^H NMR (400 MHz, DMSO, p.p.m.): 7.06 (s, 1 H), 7.44 (d, J = 8.52 Hz, 2 H), 8.01 (d, J = 8.52 Hz, 2 H); ^13^C (150 MHz, DMSO, p.p.m.): 104.5, 127.9, 129.5, 130.3, 132.8, 142.0, 162.2.

### Refinement

All H Atoms were positioned geometrically and treated as riding atoms (C—H = 0.93 Å), with U^iso^(H)=1.2 *U*_eq_(C) except H2A and H2B. The latter H atoms were located in a difference Fourier map and were refined with restrained geometry (Nardelli, 1999). The O—H distance was restained to 0.85 (6)Å and H···H distances were restained to 1.365Å thus leading to an angle of 107 Å.

### Crystal data

**Table:**

[Na(C_9_H_5_ClNOS_2_)(H_2_O)]	*F*_000_ = 576
*M**_r_* = 283.72	*D*_x_ = 1.623 Mg m^−^^3^
Orthorhombic, *P**c**a*2_1_	Mo *K*α radiation λ = 0.71073 Å
Hall symbol: P 2c -2ac	Cell parameters from 1313 reflections
*a* = 39.264 (5) Å	θ = 2.1–23.1º
*b* = 4.168 (1) Å	µ = 0.71 mm^−^^1^
*c* = 7.097 (1) Å	*T* = 300 (2) K
*V* = 1161.4 (4) Å^3^	Prism, light yellow
*Z* = 4	0.60 × 0.28 × 0.02 mm

### Data collection

**Table:**

Oxford Diffraction Xcalibur with Sapphire CCD detector diffractometer	2288 independent reflections
Radiation source: Enhance (Mo) X-ray Source	1860 reflections with *I* > 2σ(*I*)
Monochromator: graphite	*R*_int_ = 0.036
Detector resolution: 8.4012 pixels mm^-1^	θ_max_ = 26.4º
*T* = 300(2) K	θ_min_ = 3.1º
Rotation method data acquisition using ω and φ scans	*h* = −47→48
Absorption correction: multi-scan(CrysAlis RED; Oxford Diffraction, 2007)	*k* = −2→5
*T*_min_ = 0.677, *T*_max_ = 0.986	*l* = −8→8
5052 measured reflections	

### Refinement

**Table:**

Refinement on *F*^2^	Hydrogen site location: inferred from neighbouring sites
Least-squares matrix: full	H atoms treated by a mixture of independent and constrained refinement
*R*[*F*^2^ > 2σ(*F*^2^)] = 0.081	*w* = 1/[σ^2^(*F*_o_^2^) + 10.6487*P*] where *P* = (*F*_o_^2^ + 2*F*_c_^2^)/3
*wR*(*F*^2^) = 0.173	(Δ/σ)_max_ = 0.069
*S* = 1.20	Δρ_max_ = 0.61 e Å^−^^3^
2288 reflections	Δρ_min_ = −0.83 e Å^−^^3^
151 parameters	Extinction correction: none
10 restraints	Absolute structure: Flack (1983), 1009 Friedel pairs
Primary atom site location: structure-invariant direct methods	Flack parameter: 0.1 (2)
Secondary atom site location: difference Fourier map	

### Special details

**Table:**

Experimental. empirical absorption correction using spherical harmonics, implemented in SCALE3 ABSPACK scaling algorithm]
Geometry. All e.s.d.'s (except the e.s.d. in the dihedral angle between two l.s. planes) are estimated using the full covariance matrix. The cell e.s.d.'s are taken into account individually in the estimation of e.s.d.'s in distances, angles and torsion angles; correlations between e.s.d.'s in cell parameters are only used when they are defined by crystal symmetry. An approximate (isotropic) treatment of cell e.s.d.'s is used for estimating e.s.d.'s involving l.s. planes.
Refinement. Refinement of *F*^2^ against ALL reflections. The weighted *R*-factor *wR* and goodness of fit *S* are based on *F*^2^, conventional *R*-factors *R* are based on *F*, with *F* set to zero for negative *F*^2^. The threshold expression of *F*^2^ > σ(*F*^2^) is used only for calculating *R*-factors(gt) *etc*. and is not relevant to the choice of reflections for refinement. *R*-factors based on *F*^2^ are statistically about twice as large as those based on *F*, and *R*- factors based on ALL data will be even larger.

### Fractional atomic coordinates and isotropic or equivalent isotropic displacement parameters (Å^2^)

**Table:**

	*x*	*y*	*z*	*U*_iso_*/*U*_eq_	
C2	−0.1784 (2)	−0.0666 (19)	0.0522 (12)	0.0262 (19)	
C4	−0.1251 (2)	0.049 (2)	0.1720 (12)	0.028 (2)	
C5	−0.1183 (3)	0.058 (3)	−0.0110 (13)	0.049 (3)	
H5	−0.0969	0.1047	−0.0602	0.059*	
C6	−0.1010 (2)	0.136 (2)	0.3269 (12)	0.030 (2)	
C7	−0.0672 (2)	0.018 (3)	0.3149 (14)	0.041 (3)	
H7	−0.0608	−0.1245	0.2202	0.049*	
C8	−0.0438 (2)	0.122 (3)	0.4503 (16)	0.043 (3)	
H8	−0.0211	0.0592	0.4419	0.051*	
C9	−0.0540 (2)	0.314 (3)	0.5942 (14)	0.038 (3)	
C10	−0.0872 (2)	0.429 (2)	0.6068 (15)	0.037 (2)	
H10	−0.0936	0.5677	0.7031	0.044*	
C11	−0.1107 (2)	0.330 (2)	0.4701 (12)	0.030 (2)	
H11	−0.1332	0.3973	0.4781	0.036*	
N3	−0.15955 (17)	−0.031 (2)	0.2072 (9)	0.0271 (18)	
O1	−0.17113 (14)	−0.0646 (14)	0.3820 (7)	0.0230 (13)	
O2	−0.28942 (14)	0.4205 (16)	−0.0027 (9)	0.0314 (15)	
H2A	−0.302 (2)	0.266 (16)	−0.047 (12)	0.038*	
H2B	−0.299 (2)	0.587 (15)	−0.058 (12)	0.038*	
S1	−0.15330 (7)	−0.0274 (8)	−0.1467 (3)	0.0431 (7)	
S2	−0.22082 (5)	−0.1443 (5)	0.0407 (3)	0.0271 (4)	
Cl1	−0.02523 (8)	0.4352 (8)	0.7662 (5)	0.0758 (11)	
Na1	−0.25584 (8)	0.3625 (9)	0.2703 (5)	0.0320 (8)	

### Atomic displacement parameters (Å^2^)

**Table:**

	*U*^11^	*U*^22^	*U*^33^	*U*^12^	*U*^13^	*U*^23^
C2	0.034 (4)	0.021 (5)	0.023 (4)	−0.004 (3)	0.002 (4)	0.015 (5)
C4	0.030 (4)	0.022 (5)	0.033 (5)	−0.001 (4)	0.006 (3)	0.024 (4)
C5	0.042 (6)	0.076 (9)	0.031 (5)	−0.010 (6)	0.009 (4)	−0.007 (6)
C6	0.030 (4)	0.023 (5)	0.037 (5)	−0.004 (4)	−0.001 (4)	−0.002 (4)
C7	0.037 (5)	0.041 (6)	0.045 (7)	−0.004 (5)	0.003 (4)	−0.005 (5)
C8	0.027 (5)	0.034 (6)	0.067 (7)	−0.005 (5)	−0.005 (5)	0.010 (6)
C9	0.033 (5)	0.032 (6)	0.050 (7)	−0.008 (5)	−0.016 (4)	0.022 (5)
C10	0.044 (6)	0.028 (6)	0.039 (5)	−0.004 (5)	−0.003 (4)	−0.003 (4)
C11	0.031 (5)	0.024 (5)	0.034 (5)	−0.002 (4)	0.002 (4)	0.000 (4)
N3	0.021 (3)	0.035 (5)	0.026 (4)	0.006 (3)	0.001 (3)	0.007 (4)
O1	0.032 (3)	0.018 (3)	0.018 (3)	−0.002 (3)	0.006 (2)	0.000 (3)
O2	0.025 (3)	0.032 (4)	0.037 (4)	0.005 (3)	−0.008 (3)	0.003 (3)
S1	0.0447 (14)	0.0600 (19)	0.0245 (11)	−0.0061 (14)	0.0058 (11)	0.0009 (15)
S2	0.0295 (10)	0.0234 (10)	0.0283 (10)	−0.0021 (9)	−0.0025 (10)	0.0016 (12)
Cl1	0.070 (2)	0.061 (2)	0.097 (3)	−0.0061 (17)	−0.048 (2)	−0.006 (2)
Na1	0.037 (2)	0.0337 (18)	0.0253 (17)	0.0020 (17)	−0.0014 (16)	0.0000 (19)

### Geometric parameters (Å, °)

**Table:**

C2—N3	1.334 (10)	C11—H11	0.9300
C2—S2	1.699 (8)	N3—O1	1.329 (8)
C2—S1	1.729 (9)	O2—Na1	2.356 (7)
C4—C5	1.326 (13)	O2—Na1^i^	2.411 (7)
C4—N3	1.415 (10)	O2—H2A	0.88 (5)
C4—C6	1.495 (12)	O2—H2B	0.89 (5)
C5—S1	1.716 (10)	S2—Na1^ii^	2.958 (4)
C5—H5	0.9300	S2—Na1^iii^	2.961 (4)
C6—C11	1.352 (12)	S2—Na1^i^	2.998 (4)
C6—C7	1.420 (12)	S2—Na1	3.001 (4)
C7—C8	1.398 (13)	Na1—O2^iv^	2.411 (7)
C7—H7	0.9300	Na1—S2^v^	2.958 (4)
C8—C9	1.359 (14)	Na1—S2^vi^	2.961 (4)
C8—H8	0.9300	Na1—S2^iv^	2.998 (4)
C9—C10	1.389 (13)	Na1—Na1^i^	3.5780 (10)
C9—Cl1	1.738 (9)	Na1—Na1^iv^	3.5780 (10)
C10—C11	1.402 (12)	Na1—Na1^vi^	4.1680 (10)
C10—H10	0.9300	Na1—Na1^iii^	4.1680 (10)
			
N3—C2—S2	127.2 (6)	Na1^i^—S2—Na1	73.23 (9)
N3—C2—S1	110.3 (6)	O2—Na1—O2^iv^	162.1 (3)
S2—C2—S1	122.5 (5)	O2—Na1—S2^v^	106.8 (2)
C5—C4—N3	111.8 (9)	O2^iv^—Na1—S2^v^	74.03 (18)
C5—C4—C6	125.8 (8)	O2—Na1—S2^vi^	74.71 (19)
N3—C4—C6	122.1 (7)	O2^iv^—Na1—S2^vi^	87.46 (18)
C4—C5—S1	112.5 (8)	S2^v^—Na1—S2^vi^	91.06 (13)
C4—C5—H5	123.8	O2—Na1—S2^iv^	115.3 (2)
S1—C5—H5	123.8	O2^iv^—Na1—S2^iv^	82.42 (17)
C11—C6—C7	121.1 (8)	S2^v^—Na1—S2^iv^	88.82 (11)
C11—C6—C4	121.3 (8)	S2^vi^—Na1—S2^iv^	169.51 (13)
C7—C6—C4	117.6 (8)	O2—Na1—S2	83.24 (19)
C8—C7—C6	117.7 (9)	O2^iv^—Na1—S2	95.48 (18)
C8—C7—H7	121.2	S2^v^—Na1—S2	169.51 (13)
C6—C7—H7	121.2	S2^vi^—Na1—S2	88.70 (11)
C9—C8—C7	120.4 (9)	S2^iv^—Na1—S2	89.52 (12)
C9—C8—H8	119.8	O2—Na1—Na1^i^	41.94 (16)
C7—C8—H8	119.8	O2^iv^—Na1—Na1^i^	124.6 (2)
C8—C9—C10	121.9 (9)	S2^v^—Na1—Na1^i^	133.11 (9)
C8—C9—Cl1	120.4 (8)	S2^vi^—Na1—Na1^i^	52.77 (9)
C10—C9—Cl1	117.7 (9)	S2^iv^—Na1—Na1^i^	132.39 (9)
C9—C10—C11	118.1 (10)	S2—Na1—Na1^i^	53.34 (9)
C9—C10—H10	120.9	O2—Na1—Na1^iv^	152.5 (2)
C11—C10—H10	120.9	O2^iv^—Na1—Na1^iv^	40.77 (15)
C6—C11—C10	120.7 (9)	S2^v^—Na1—Na1^iv^	52.85 (8)
C6—C11—H11	119.6	S2^vi^—Na1—Na1^iv^	119.08 (11)
C10—C11—H11	119.7	S2^iv^—Na1—Na1^iv^	53.43 (8)
O1—N3—C2	124.6 (7)	S2—Na1—Na1^iv^	118.65 (11)
O1—N3—C4	121.1 (6)	Na1^i^—Na1—Na1^iv^	165.3 (2)
C2—N3—C4	114.2 (7)	O2—Na1—Na1^vi^	84.11 (18)
Na1—O2—Na1^i^	97.3 (2)	O2^iv^—Na1—Na1^vi^	84.24 (18)
Na1—O2—H2A	123 (6)	S2^v^—Na1—Na1^vi^	45.98 (8)
Na1^i^—O2—H2A	97 (6)	S2^vi^—Na1—Na1^vi^	46.04 (8)
Na1—O2—H2B	134 (6)	S2^iv^—Na1—Na1^vi^	134.80 (8)
Na1^i^—O2—H2B	96 (6)	S2—Na1—Na1^vi^	134.74 (8)
H2A—O2—H2B	100 (6)	Na1^i^—Na1—Na1^vi^	90.0
C5—S1—C2	91.0 (4)	Na1^iv^—Na1—Na1^vi^	90.0
C2—S2—Na1^ii^	117.9 (3)	O2—Na1—Na1^iii^	95.89 (18)
C2—S2—Na1^iii^	124.2 (3)	O2^iv^—Na1—Na1^iii^	95.76 (18)
Na1^ii^—S2—Na1^iii^	74.38 (9)	S2^v^—Na1—Na1^iii^	134.02 (8)
C2—S2—Na1^i^	101.3 (3)	S2^vi^—Na1—Na1^iii^	133.96 (8)
Na1^ii^—S2—Na1^i^	88.82 (11)	S2^iv^—Na1—Na1^iii^	45.20 (8)
Na1^iii^—S2—Na1^i^	134.38 (7)	S2—Na1—Na1^iii^	45.26 (8)
C2—S2—Na1	106.8 (3)	Na1^i^—Na1—Na1^iii^	90.0
Na1^ii^—S2—Na1	134.38 (7)	Na1^iv^—Na1—Na1^iii^	90.0
Na1^iii^—S2—Na1	88.70 (11)	Na1^vi^—Na1—Na1^iii^	180.000 (1)
			
N3—C4—C5—S1	0.3 (14)	Na1^i^—O2—Na1—S2	−39.9 (2)
C6—C4—C5—S1	−174.8 (8)	Na1^i^—O2—Na1—Na1^iv^	175.14 (18)
C5—C4—C6—C11	133.0 (12)	Na1^i^—O2—Na1—Na1^vi^	96.6 (2)
N3—C4—C6—C11	−41.7 (13)	Na1^i^—O2—Na1—Na1^iii^	−83.4 (2)
C5—C4—C6—C7	−45.0 (15)	C2—S2—Na1—O2	129.3 (3)
N3—C4—C6—C7	140.3 (10)	Na1^ii^—S2—Na1—O2	−38.8 (3)
C11—C6—C7—C8	−3.5 (15)	Na1^iii^—S2—Na1—O2	−105.24 (18)
C4—C6—C7—C8	174.5 (9)	Na1^i^—S2—Na1—O2	32.28 (18)
C6—C7—C8—C9	4.0 (16)	C2—S2—Na1—O2^iv^	−32.8 (3)
C7—C8—C9—C10	−3.7 (16)	Na1^ii^—S2—Na1—O2^iv^	159.16 (19)
C7—C8—C9—Cl1	178.3 (8)	Na1^iii^—S2—Na1—O2^iv^	92.68 (18)
C8—C9—C10—C11	2.7 (15)	Na1^i^—S2—Na1—O2^iv^	−129.80 (19)
Cl1—C9—C10—C11	−179.2 (7)	C2—S2—Na1—S2^v^	−34.2 (10)
C7—C6—C11—C10	2.7 (14)	Na1^ii^—S2—Na1—S2^v^	157.7 (7)
C4—C6—C11—C10	−175.2 (8)	Na1^iii^—S2—Na1—S2^v^	91.2 (9)
C9—C10—C11—C6	−2.2 (14)	Na1^i^—S2—Na1—S2^v^	−131.3 (9)
S2—C2—N3—O1	3.1 (13)	C2—S2—Na1—S2^vi^	54.6 (3)
S1—C2—N3—O1	−176.2 (7)	Na1^ii^—S2—Na1—S2^vi^	−113.52 (17)
S2—C2—N3—C4	−177.0 (7)	Na1^iii^—S2—Na1—S2^vi^	180.0
S1—C2—N3—C4	3.7 (10)	Na1^i^—S2—Na1—S2^vi^	−42.48 (9)
C5—C4—N3—O1	177.3 (9)	C2—S2—Na1—S2^iv^	−115.1 (3)
C6—C4—N3—O1	−7.4 (14)	Na1^ii^—S2—Na1—S2^iv^	76.8 (2)
C5—C4—N3—C2	−2.7 (13)	Na1^iii^—S2—Na1—S2^iv^	10.34 (13)
C6—C4—N3—C2	172.7 (8)	Na1^i^—S2—Na1—S2^iv^	147.86 (14)
C4—C5—S1—C2	1.5 (10)	C2—S2—Na1—Na1^i^	97.1 (3)
N3—C2—S1—C5	−2.9 (8)	Na1^ii^—S2—Na1—Na1^i^	−71.04 (19)
S2—C2—S1—C5	177.7 (6)	Na1^iii^—S2—Na1—Na1^i^	−137.52 (9)
N3—C2—S2—Na1^ii^	−135.5 (7)	C2—S2—Na1—Na1^iv^	−68.2 (3)
S1—C2—S2—Na1^ii^	43.7 (6)	Na1^ii^—S2—Na1—Na1^iv^	123.7 (2)
N3—C2—S2—Na1^iii^	−46.0 (9)	Na1^iii^—S2—Na1—Na1^iv^	57.21 (18)
S1—C2—S2—Na1^iii^	133.2 (4)	Na1^i^—S2—Na1—Na1^iv^	−165.27 (19)
N3—C2—S2—Na1^i^	129.8 (8)	C2—S2—Na1—Na1^vi^	54.6 (3)
S1—C2—S2—Na1^i^	−51.0 (6)	Na1^ii^—S2—Na1—Na1^vi^	−113.52 (17)
N3—C2—S2—Na1	54.1 (8)	Na1^iii^—S2—Na1—Na1^vi^	180.0
S1—C2—S2—Na1	−126.7 (5)	Na1^i^—S2—Na1—Na1^vi^	−42.48 (9)
Na1^i^—O2—Na1—O2^iv^	47.1 (12)	C2—S2—Na1—Na1^iii^	−125.4 (3)
Na1^i^—O2—Na1—S2^v^	137.05 (17)	Na1^ii^—S2—Na1—Na1^iii^	66.48 (17)
Na1^i^—O2—Na1—S2^vi^	50.6 (2)	Na1^i^—S2—Na1—Na1^iii^	137.52 (9)
Na1^i^—O2—Na1—S2^iv^	−126.10 (17)		

Symmetry codes: (i) −*x*−1/2, *y*, *z*−1/2; (ii) −*x*−1/2, *y*−1, *z*−1/2; (iii) *x*, *y*−1, *z*; (iv) −*x*−1/2, *y*, *z*+1/2; (v) −*x*−1/2, *y*+1, *z*+1/2; (vi) *x*, *y*+1, *z*.

### Hydrogen-bond geometry (Å, °)

**Table:**

*D*—H···*A*	*D*—H	H···*A*	*D*···*A*	*D*—H···*A*
O2—H2A···O1^i^	0.88 (5)	1.80 (5)	2.675 (8)	175 (9)
O2—H2A···N3^i^	0.88 (5)	2.61 (6)	3.434 (9)	156 (8)
O2—H2B···O1^vii^	0.89 (5)	1.91 (6)	2.770 (8)	164 (9)

Symmetry codes: (i) −*x*−1/2, *y*, *z*−1/2; (vii) −*x*−1/2, *y*+1, *z*−1/2.
